# The Use of Retinoids for the Prevention and Treatment of Skin Cancers: An Updated Review

**DOI:** 10.3390/ijms232012622

**Published:** 2022-10-20

**Authors:** Brandon Ramchatesingh, Amelia Martínez Villarreal, Domenico Arcuri, François Lagacé, Samy Abu Setah, Fadi Touma, Faris Al-Badarin, Ivan V. Litvinov

**Affiliations:** 1 Division of Experimental Medicine, McGill University, Montreal, QC H4A 3J1, Canada; 2 Faculty of Medicine and Health Sciences, McGill University, Montreal, QC H4A 3J1, Canada; 3 Division of Dermatology, McGill University Health Center, Montreal, QC H4A 3J1, Canada; 4 Faculté de Médicine, Université Laval, Québec, QC G1V 0V6, Canada

**Keywords:** retinoids, chemoprevention, chemotherapy, cutaneous squamous cell carcinoma, basal cell carcinoma, cutaneous T-cell lymphoma, Kaposi’s sarcoma, alitretinoin, acitretin, bexarotene

## Abstract

Retinoids are natural and synthetic vitamin A derivatives that are effective for the prevention and the treatment of non-melanoma skin cancers (NMSC). NMSCs constitute a heterogenous group of non-melanocyte-derived skin cancers that impose substantial burdens on patients and healthcare systems. They include entities such as basal cell carcinoma and cutaneous squamous cell carcinoma (collectively called keratinocyte carcinomas), cutaneous lymphomas and Kaposi’s sarcoma among others. The retinoid signaling pathway plays influential roles in skin physiology and pathology. These compounds regulate diverse biological processes within the skin, including proliferation, differentiation, angiogenesis and immune regulation. Collectively, retinoids can suppress skin carcinogenesis. Both topical and systemic retinoids have been investigated in clinical trials as NMSC prophylactics and treatments. Desirable efficacy and tolerability in clinical trials have prompted health regulatory bodies to approve the use of retinoids for NMSC management. Acceptable off-label uses of these compounds as drugs for skin cancers are also described. This review is a comprehensive outline on the biochemistry of retinoids, their activities in the skin, their effects on cancer cells and their adoption in clinical practice.

## 1. Introduction

In the early 20th century, retinol (commonly known as vitamin A) was isolated and characterized as an essential nutrient for human health. Retinol deficiencies were associated with visual impairments and precancerous squamous metaplasia in multiple organs [1,2]. With time, retinol would gain appreciation for its critical roles in regulating skin physiology [3,4,5]. Natural and synthetic analogues of retinol, termed “retinoids”, were compounded into systemic and topical agents and studied as treatments for various skin diseases [6]. Systemic retinoids were first used successfully as a treatment for acne vulgaris in 1943 by Straumfjord [7]. Stüttgen and Kligman would later pioneer topical retinoids for the treatment of keratinizing disorders and other conditions [8,9]. Both systemic and topical retinoids are approved for clinical uses by the United States Food and Drug Administration (FDA) as monotherapies and as components of combination therapy for several skin disorders [10,11]. At the time of publication, retinoids are indicated for conditions such as pustular psoriasis, acne vulgaris, fine wrinkling, and hyperpigmentation among other uses [10,11]. Provided their well-established and wide-ranging effects on human skin, retinoids are readily considered for other, off-label uses in dermatology clinics [10,11].

The retinoid signaling pathway has been the subject of rigorous biochemical characterization, and has emerged as an appealing target for the development of cancer therapeutics [12]. Retinoids exert control over a range of processes central to cancer formation and maintenance, such as proliferation, differentiation and apoptosis among others [12]. For several solid and hematological cancers, retinoids have been contemplated and implemented as prophylactics and as therapeutics [13].

Topical and systemic retinoids are effective prophylactics and therapeutics for several types of non-melanoma skin cancers (NMSCs) [14]. NMSC is an umbrella term for all cutaneous malignancies that are not derived from melanocytes [15,16,17,18,19,20,21]. Keratinocyte carcinoma (KC) is the most prevalent subclass of NMSC [22,23,24,25]. KCs arise from subpopulations of interfollicular epidermal keratinocytes and hair follicle stem cells [26,27,28,29]. Basal cell carcinoma (BCC) and cutaneous squamous cell carcinoma (cSCC) are the two types of KC, representing the first and fifth most prevalent human malignancies worldwide [30,31,32,33]. Prominent risk factors for the development of KC include exposure to ultraviolet (UV) or ionizing radiation, fair skin (Fitzpatrick phototype I-III skin tones), age, chemical exposures (e.g., arsenic), certain drugs (e.g., BRAF inhibitors, cyclosporin, voriconazole, hydrochlorothiazide) and viruses such as human papillomavirus (HPV) [34]. cSCC can develop from erythematous scaly papules consisting of dysplastic keratinocytes on photodamaged skin, known as actinic keratoses [35]. Immunosuppression (e.g., Solid Organ Transplant Recipients [SOTR] or others prescribed immunosuppressants) is another prominent risk factor, increasing the risk of developing cSCC by up to 65-fold and the risk of developing BCC by up to 10-fold [36]. Inherited genodermatoses, such as Gorlin-Goltz (nevoid BCC syndrome; NBCCS) syndrome and xeroderma pigmentosum (XP), amongst others, confer predispositions to BCC and cSCC formation [31,32,33,37]. The prospect of using retinoids to treat and prevent KC has been investigated thoroughly.

Retinoids have also been investigated as drugs for other skin cancers. Most notably, cutaneous T-cell lymphomas (CTCL) and Kaposi’s sarcoma (KS) [15]. CTCL constitutes a heterogenous group of extranodal non-Hodgkin’s lymphomas that arise from skin-resident or skin-homing T lymphocytes [38,39,40,41,42]. Mycosis Fungoides (MF) and the aggressive leukemic variant, Sézary syndrome (SS), are the two most commonly recognized CTCL subtypes, accounting for ~55% of cases [43,44,45,46,47,48,49,50]. Other subtypes include anaplastic large cell lymphoma and CD30^+^ lymphoproliferative disorders, among others [43]. Retinoids can modulate several aspects of the cutaneous immune landscape, regulating the biology of CTCL cells and their microenvironments, while also having a direct impact on malignant T lymphocytes [51].

KS arises from endothelial cells that have been transformed by Kaposi’s Sarcoma Herpesvirus (KSHV; human herpesvirus-8; HHV-8) [52]. 95% of KS tumors contain KSHV integration [53]. In North America, KS is most often associated with HIV-AIDS, iatrogenic immunosuppression or advanced age, although other subtypes are described and include classic (Mediterranean) and African endemic [52]. Virus-encoded genes, including viral interleukin-6 (IL-6), have essential roles in KS pathogenesis [54]. Other NMSCs include Merkel Cell Carcinoma, dermatofibrosarcoma protuberans, trichoepitheliomas and other rare entities. While retinoids have been examined to some degree for the treatment of these malignancies, the use of these drugs to prevent or treat these cancers is not suggested by biological or clinical evidence.

NMSCs impose considerable burdens on patients due to their direct morbidity and mutilating cosmetic impacts. Although the majority of these cancers can be managed with surgery and ablation, there is a persistent need to optimize approaches to cancer prevention and treatment. Optimizing current interventions and improving upon existing algorithms to support patient wellbeing and alleviate the strain on care providers is imperative. Retinoids have been widely investigated as therapeutics for skin cancers, and have been a staple in dermatology clinics following decades of research. Guidelines for the use of retinoids for skin cancer management are continually updated based on emerging research. This review provides a comprehensive summary and update on the biological basis for retinoid action in the skin, and the experimental and clinical use of retinoids for the prevention and treatment of skin cancers.

## 2. The Biochemistry of Retinoids and Retinoid Receptors

### 2.1. The Structures and Classifications of Retinoid Products

**Figure 1:**
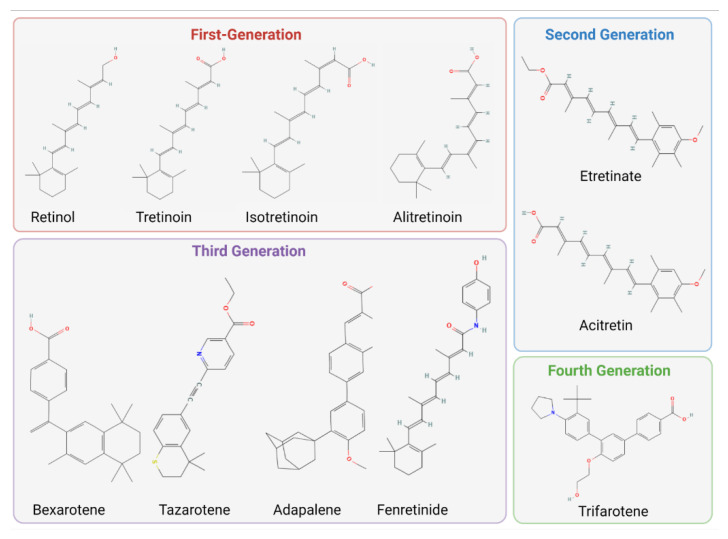
Chemical structures of clinically used retinoids from each generation. 2D-Structures taken from Pubchem.com. Available online: https://pubchem.ncbi.nlm.nih.gov/compound/Retinol#section=2D-Structure (accessed on 19 September 2022); https://pubchem.ncbi.nlm.nih.gov/compound/Tretinoin#section=2D-Structure (accessed on 19 September 2022); https://pubchem.ncbi.nlm.nih.gov/compound/Alitretinoin (accessed on 19 September 2022); https://pubchem.ncbi.nlm.nih.gov/compound/Isotretinoin#section=2D-Structure (accessed on 19 September 2022); https://pubchem.ncbi.nlm.nih.gov/compound/Etretinate (accessed on 19 September 2022); https://pubchem.ncbi.nlm.nih.gov/compound/Acitretin#section=2D-Structure (accessed on 19 September 2022); https://pubchem.ncbi.nlm.nih.gov/compound/Bexarotene#section=2D-Structure (accessed on 19 September 2022); https://pubchem.ncbi.nlm.nih.gov/compound/Tazarotene#section=2D-Structure (accessed on 19 September 2022); https://pubchem.ncbi.nlm.nih.gov/compound/Adapalene#section=2D-Structure (accessed on 19 September 2022); https://pubchem.ncbi.nlm.nih.gov/compound/Fenretinide#section=2D-Structure (accessed on 19 September 2022); https://pubchem.ncbi.nlm.nih.gov/compound/Trifarotene#section=2D-Structure (accessed on 19 September 2022). Figure generated with Biorender.com.

Structurally, retinoids are defined by four isoprene subunits with head-to-tail structure containing polar end-group modifications [55]. There are currently four generations of retinoids that possess distinct structural hallmarks that modify their mechanisms of action. First-generation retinoids consist of naturally derived monoaromatic compounds such as all-trans retinol (also known as retinol; vitamin A; ATRol), and its bioactive derivatives: all-trans-retinoic acid (also known as tretinoin or ATRA), 9-cis-retinoic acid (also known as 9-cis-RA or alitretinoin) and 13-cis-retinoic acid (also known as 13-cis-RA or isotretinoin) [56,57]. The second, third and fourth generations consist of synthetic compounds. Second-generation retinoids contain monoaromatic ring structures, and include etretinate and its bioactive metabolite, acitretin [57]. Third generation retinoids were synthesized with rigid polyaromatic ring structures to improve uptake and stability [57]. Bexarotene, tazarotene, fenretinide and adapalene are third generation retinoids [57]. A fourth-generation retinoid, trifarotene, was designed to limit off-target effects by targeting a specific retinoic acid signaling pathways with higher selectivity [57,58]. Topical and systemic formulations of these compounds have been developed for various uses in dermatology. Retinoid structures are visualized in Figure 1.

### 2.2. Molecular Mechanisms of Canonical Retinoid Signaling

**Figure 2:**
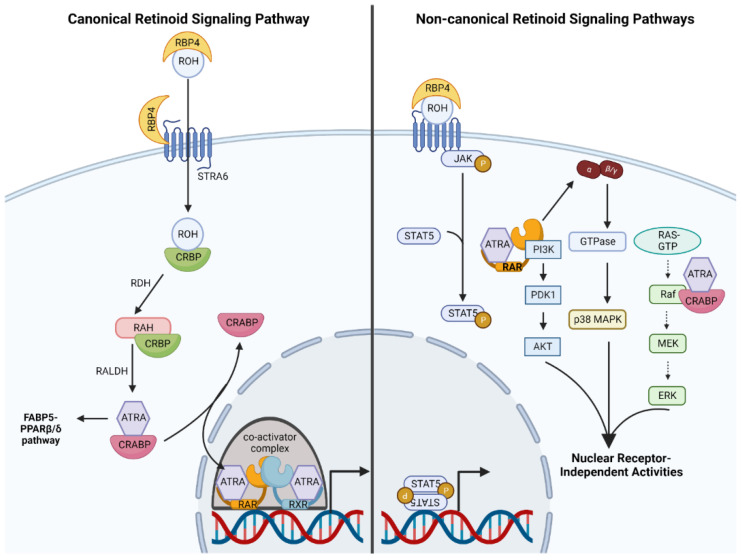
Canonical and non-canonical retinoid signaling pathways. (**Left**) retinol (ROH) binds RBP4 in circulation. RBP4 binds STRA6 which mediates retinol import into cells. CRBP binds ROH within the cell and holds the retinoid during enzymatic conversions (via retinol dehydrogenase [RDH] and retinaldehyde dehydrogenase [RALDH]) to ATRA. CRABP enzymes bind ATRA and deliver ATRA to RAR-RXR heterodimers bound to DNA at the RARE element. Co-activator complexes enable transcriptional activation at the RARE element. (**Right**) select examples of non-canonical retinoid pathways mediating nuclear receptor-independent signaling via activation of the JAK-STAT axis, PI3K signaling, activation of G protein signaling to p38 MAPK and modulation of the Ras-Raf-MEK-ERK Cascade. Figure created using Biorender.com.

Retinoids signal through canonical and non-canonical signaling pathways. Retinol’s mechanism of action is a representative model of canonical signaling [59]. Systemic retinol is absorbed in the intestine, incorporated into chylomicrons and transported through lymphatic circulation [60]. Systemic retinol also enters the bloodstream but requires carrier proteins, such as Retinol Binding Protein 4 (RBP4), to facilitate transit through blood plasma to target cells [61]. On the other hand, topically applied retinol undergoes percutaneous absorption, bypassing systemic circulation to access skin cells directly [62]. Retinol is imported into target cells through a channel formed by Stimulated by Retinoic Acid 6 (STRA6) [63]. Retinol-bound RBP4 binds STRA6, embedded in the plasma membrane. Retinol is then transferred across the membrane, leaving RBP4 outside the cell. Inside the cell, retinol binds cellular retinol binding protein (CRBP) [64] which facilitates intracellular trafficking, and enzymatic conversion of retinol to retinaldehyde (via retinol dehydrogenase). Retinaldehyde dehydrogenases convert retinaldehyde to bioactive retinoic acids (ATRA, 9-cis RA or 13-cis RA) [59]. Retinoic acids in the cytoplasm bind intracellular carriers: cellular retinoic acid binding proteins I/II (CRABPI/II) or fatty acid binding protein 5 (FABP5) [65]. CRABPI/II transfers ATRA to heterodimers formed by retinoic acid receptors (RARs) and retinoid X receptors (RXRs) (Figure 2). FABP5 transfers ATRA to peroxisome proliferator-activated receptor β/δ (PPARβ/δ) heterodimerized with RXRs [66]. RARs and PPARs are type II nuclear receptors that form heterodimers with RXRs, and act as ligand-dependent transcription factors [67,68]. It is through activating nuclear receptors and modulating transcription of target genes that retinoids induce their canonical signaling functions [67]. The ratio of CRABPII to FABP5 within a cell was hypothesized to determine whether PPAR β/δ- or RAR-mediated signaling predominates [66]. These signaling cascades have been proposed to mediate opposite effects (proliferation vs. growth arrest and survival vs. apoptosis) [66]. The hypothesis that these signaling cascades have opposing effects has been challenged by contradictory evidence [69]. CRABPII has more restricted expression compared to CRABPI, and it is the predominant form in the skin [70]. FAPB5 is also expressed in healthy epidermis but its expression is elevated in diseased/inflamed skin [71,72]. Provided that the RAR-RXR signaling axis is better characterized in the context of NMSC pathogenesis and treatment, it is described further.

There are three RAR and RXR isoforms, denoted as α, β and γ [68]. Multiple stereoisomers exist for each of the three isoforms [68]. Within the skin the predominant RAR isoform is RARγ and the predominant RXR isoform is RXRα [59]. RARγ-RXRα is, therefore, believed to be the principal heterodimer in the skin [59]. Transcriptional activation by retinoids is outlined in an extensive review by Bastien and Rochette-Egly [73]. In brief, in the absence of a ligand, RAR-RXR heterodimers are bound to retinoic acid response elements (RARE) in the DNA and recruit a corepressor complex that includes a histone deacetylase (HDAC) [12]. Upon binding to a ligand, RAR-RXR heterodimers undergo a conformational change allowing for transcriptional de-repression and recruitment of a co-activator complex containing a histone acetyltransferase (HAT) that renders chromatin accessible at the RARE element [74]. Transcriptional targets of retinoid signaling vary according to the cell types and the presence of competing/opposing signals. Retinoid signaling is terminated through a variety of mechanisms including proteasomal degradation of retinoid receptors, degradation via CYP26 enzymes and retinoid export to the extracellular space [12,73].

The signaling mechanisms followed by synthetic retinoids deviate in various ways from retinol’s mechanism of action. Etretinate is the prodrug of acitretin [75]. Acitretin exhibits low affinity for RAR-RXR heterocomplexes but a high affinity for CRABPI/II [76]. Acitretin displaces endogenous RA from CRABP, accelerating the transfer of RA to RAR-RXR complexes, thereby potentiating retinoid signaling indirectly [77,78]. Third-generation retinoids exist in their bioactive forms, and bind directly to specific RARs and RXRs [79]. For example, bexarotene acts as a “rexinoid”: a selective pan-RXR agonist [80], while tazarotene and adapalene exhibit higher affinity for RARβ/γ [81]. Fenretinide, exhibits low affinity for RARs and RXRs [82,83]. RARE activation likely plays a minor role in the mechanism of fenretinide action [84]. The fourth-generation retinoid, trifarotene, is a highly selective RARγ agonist with limited off-target activity [58]. As these agents are known to have different clinical benefits/effects in dermatology practice (e.g., isotretinoin is effective for the treatment of acne conglobate, acitretin is more effective for the treatment of psoriasis, trifarotene is FDA-approved for acne vulgaris, etc.) it is clear that biologic impact of these agents on skin differs significantly. 

### 2.3. Non-Canonical Retinoid Signaling: An Emerging Phenomenon 

Retinoids also elicit non-canonical pathways, independent of transcriptional activation by nuclear receptors, and modulate crosstalk with other signaling networks (Figure 2) [59]. Albeit an emerging area of research, these non-canonical pathways are physiologically relevant and may be important for promoting anti-cancer effects. ATRA-bound CRABPI has emerged as an important signaling factor controlling the mitogen activated protein (MAP) kinase pathway [85]. Specifically, ATRA-bound CRABPI binds the Ras binding domain of Raf, mediating downstream repression of extracellular signaling regulated kinase 1 and 2 (ERK1/2) independent of growth factor signaling [85]. RAR and RXR can also modulate the PI3 kinase-Akt-mTOR signaling pathway [86,87]. A recent report detailed a noteworthy paradigm whereby truncated RXRα (as a result of mutation) forms a homotetrameric complex that activates PI3K signaling [87]. Ligand-bound RARα may also bind and potentiate activity of the p110 catalytic subunit of PI3K [86]. The Janus Kinase (JAK)/signal transducer and activator of transcription (STAT) signaling pathway is activated through a scaffold formed by ligand-bound RBP4 and STRA6 [88]. ATRA-bound RARα localized in lipid rafts can mediate signaling through G protein αq and p38 MAP kinase [89]. Although non-genomic mechanisms are a recently described and emerging phenomenon, it is clear that these pathways are important to skin physiology and the use of these compounds as cancer therapeutics. Figure 2 summarizes the canonical and some non-canonical retinoid signaling pathways.

## 3. The Biological Effects of Retinoids in the Skin

The effects of retinoid signaling on skin physiology have been studied extensively [90]. Retinoids influence diverse aspects of the biology of epidermal keratinocytes, dermal fibroblasts, skin-resident immune cells, and vascular endothelial cells [59]. Examples of processes modulated by retinoids include cell turnover, differentiation, barrier functions, immunity, vascular remodeling and wound healing [57,91,92,93,94]. The activities of retinoids in healthy skin are harnessed to repress cancer formation.

### 3.1. Retinoids Control Epidermal Maturation and Turnover

Retinoids stimulate the process of epidermal turnover [57]. Epidermal turnover is the homeostatic tissue renewal process whereby differentiated keratinocytes (corneocytes) undergo desquamation, and are simultaneously replaced by keratinocytes that differentiate and migrate from basal to superficial layers [95,96]. This process is a delicate balance between basal cell proliferation, differentiation, cell death by cornification and desquamation [96]. This effect of retinoids is important for the treatment of diseases of keratinization such as psoriasis, lichen planus, various ichthyosis and to ameliorate fine wrinkling of the skin.

Physiological retinoid signaling inhibits proliferation of epidermal keratinocytes. Ablation of RARs or transport proteins in epidermal keratinocytes, either in vitro or in vivo, results in basal keratinocyte hyperproliferation, while overexpression promotes proliferation arrest [97,98,99,100]. In contrast, high-dose, pharmacological retinoid signaling promotes keratinocyte proliferation. Topical or systemic retinoids, including precursors such as β-carotene or retinaldehyde, induced epidermal hyperplasia in animal models by increasing the thickness of the spinous and granular layers of the epidermis [101,102,103] These effects have been observed in vitro and in vivo, in human and animal subjects, treated with several types of retinoids [102,103,104,105,106,107]. Upregulation of heparin-binding epidermal-growth factor-like (HB-EGF) signaling and activation of FABP5-PPAR β/γ have been proposed as mechanisms underlying the growth-promoting effects of high-dose retinoids [66,108,109]. On the other hand, several studies provide compelling evidence that high-dose retinoids can induce growth arrest [106,110,111,112,113,114,115]. Several growth suppressive mechanisms have been demonstrated, including the activation of tumor suppressor genes like tazarotene-inducible gene 3 (TIG-3), induction of DNA damage and S phase cell cycle arrest, and repression of proliferation-promoting STAT signaling [113,114,115]. The discrepancy between these experimental observations has been attributed to context dependency. Retinoid signaling acts in concert with many different signals to either support proliferation or induce arrest [116]. As such, retinoids have been proposed to amplify the effect of other signals. Differences between experimental models (e.g., cell lines) and agonist-specific effects (e.g., pathways induced by specific compounds) can also determine the response to retinoids.

Retinoids modulate epidermal differentiation. Animal models and human cases of retinol deficiency provided the first evidence of this [117]. Squamous metaplasia, keratinization defects and other skin abnormalities were reported in cases of retinoid deficiency [3,117]. In embryogenesis and throughout life, a physiological level of retinoid signaling regulates epidermal differentiation [98]. Disturbances in the retinoid signaling pathway impair epidermal differentiation [98,118,119]. Both in vitro and in vivo, pharmacological doses of retinoid treatment suppress various stages of epidermal differentiation. Early stage differentiation is impaired, as indicated by the repression of basal cytokeratins 5 and 14, as well as suprabasal keratins 1 and 10 [92,120]. The final stages of epidermal differentiation, during which time the cornified envelope forms, is also suppressed [121,122,123]. Expression of mucosal keratins (keratin 13) and luminal keratins (keratin 18) are induced by retinoid treatment [120,124]. It has been proposed that high doses of retinoids may support a mucosal differentiation program while suppressing a squamous differentiation program [124]. Mechanistically, this repression may involve regulation of p63, a central transcription factor implicated in terminal differentiation of squamous epithelia [125]. Δp63α is the predominant isoform of p63, that is expressed in basal layer keratinocyte stem cells [126]. As epidermal differentiation progresses, Δp63α expression in keratinocytes decreases [127]. Retinoic acid treatment can inhibit this downregulation, preventing the differentiation-associated decrease in this transcription factor [128]. The impact of retinoids on differentiation likely acts in concert with other signals [116]. For example, retinoids stimulate synthesis of terminal differentiation genes when applied to differentiating keratinocytes cultured in the presence of high calcium in vitro [129]. These properties make retinoids invaluable in the treatment of a variety of papulosquamous conditions affecting the skin/mucosa (lichen planus, psoriasis, Darier disease, etc.) and acquired/inherited disorders of keratinization (ichthyoses, palmoplantar keratodermas, etc.)

Retinoid-induced keratinocyte apoptosis is documented and involves both canonical and non-canonical signaling pathways [130,131]. Keratinocytes treated with ATRA in vitro undergo apoptosis, increasing expression of p53 as well as the expression of caspase 3, 7, 8 and 9 mRNAs and proteins [130,131]. Other apoptosis-related factors upregulated by retinoid signaling include Fas and BH3 interacting domain death agonist (BID) [92]. Beyond the transcriptional effects, Louafi et al. reported that the induction of keratinocyte apoptosis involved nongenomic inhibition of insulin-like growth factor II (IGF2) signaling [132]. Selective RAR- and RXR-specific retinoids are capable of inducing keratinocyte apoptosis [131]. Tazarotene promotes apoptosis of immortalized keratinocytes via transcriptional upregulation of p73, a tumor-suppressive p53 homologue [133]. Although cell death is intimately tied to terminal differentiation in the skin and the process of cornification, the induction of apoptosis by retinoids is likely independent of the terminal differentiation program. In one study, retinoids repressed expression of keratinocyte differentiation markers while concurrently inducing apoptosis under differentiating culture conditions [131,134]. Caspase-14, believed to be a central effector of cornified cell death and terminal differentiation, is also repressed by retinoid treatment in murine skin even as cells undergo apoptosis [135,136]. This may suggest that the apoptotic program initiated by retinoid signaling is independent of the epidermal differentiation program.

The complex, and at times opposing, effects of retinoids on epidermal keratinocytes may be summarized as follows. A physiological level of retinoid signaling is necessary for suppressing proliferation and promoting normal differentiation/keratinization. This physiological level of retinoid signaling is tumor suppressive. Pharmacological doses of retinoid signaling act in concert with other signals within the skin. High doses of retinoids are shown to (1) promote proliferation and epidermal thickening (2) inhibit squamous cell differentiation programs and cornification and (3) promote apoptosis. As highlighted above, these effects make retinoids useful for the treatment of keratinization disorders, KC and other cancers and their precursor lesions. The reported contradictory effects of retinoids on proliferation brings into question the use of these compounds as therapeutic agents. Nonetheless, it is evident that retinoids do exert an anti-tumorigenic effect within the skin. This may involve a predominance of anti-tumorigenic effects (e.g., apoptosis and cell cycle arrest) over the pro-tumorigenic effect (e.g., proliferation). One mechanism that has been proposed is the predominance of RAR-RXR pathway (anti-tumorigenic) over the PPARβ/γ-RXR pathway (pro-tumorigenic) [66]. However, this explanation has been controversial. Another explanation is that the microenvironment within the skin, which cannot be replicated in its entirety in an experimental model, provides a molecular context that supports retinoids’ anti-tumorigenic effects. Importantly, dermatology patients receive retinoid treatments for years and decades (e.g., acitretin for psoriasis treatment) without a documented increased risk of malignancies.

### 3.2. Retinoids Influence the Immune Landscape of the Skin

Innate immune effectors within the skin, including dendritic cells and Langerhans cells, are subject to regulation by retinoids [137,138]. Topical ATRA treatment prevents decrease in Langerhans and dendritic cell density in murine skin exposed to UV radiation or chemical carcinogens [138]. Skin resident and skin-homing T lymphocytes are also subject to regulation by retinoids. Retinoids promote apoptosis of T lymphocytes and can regulate how circulating lymphocytes home to the skin by regulating the expression of cell-surface adhesion molecules in the epidermis and the infiltrating T cells [139,140]. The detailed immune modulating effects of retinoids are useful in the treatment of inflammatory skin diseases such as acne and rosacea.

In addition to modifying cellular immunity within the skin, retinoids can support cell-intrinsic immune defenses by limiting replication of some viruses. Human Papilloma Virus (HPV) infection can induce the formation of cSCCs, driven by the E6 and E7 viral oncoproteins [141]. In keratinocytes infected with HPV-16, retinoid treatment repressed transcription of E6 and E7, impairing transformation [142,143,144,145]. Additionally, HPV-16 infected keratinocytes were sensitized to retinoid-induced growth arrest and keratinization changes compared to HPV-negative keratinocytes [142]. Likewise, retinoids inhibit replication of KSHV, the primary causative agent in KS, in endothelial and epithelial cells in vitro [146]. Furthermore, retinoids support healthy barrier defenses, by regulating lipid synthesis and junctional complexes [147]. Trifarotene, for example, drives the expression of aquaporin-3 and peptidyl arginase deaminase, which support skin hydration and the integrity of the skin barrier [148].

### 3.3. Skin Structure and Vascularization Are Regulated by Retinoid Signaling

The complex architecture of the skin is also modified by retinoids. In keratinocytes, retinoids regulate the activity of enzymes, such as collagenases and components of the urokinase plasminogen activator system, which function to inhibit matrix metalloproteases (MMP) [149,150]. Recently, trifarotene has been shown through gene expression studies to decreased expression of MMP genes in keratinocytes [148]. Retinoid signaling also drives turnover of intercellular adhesions, including claudins and corneodesmosomes [147,151], thereby permitting mobilization of keratinocytes within the epidermis and allowing for eventual desquamation.

Within the dermis and hypodermis, retinoids control vascularization. Retinoids decrease the expression and secretion of vascular endothelial growth factor (VEGF) by keratinocytes [152]. By decreasing VEGF produced by the keratinocytes, retinoids restrict dermal angiogenesis [152,153]. ATRA pre-treatment can also attenuate UV-induced VEGF production by keratinocytes, attributed to the downregulation of the MAPK/ERK pathway [153].

**Figure 3:**
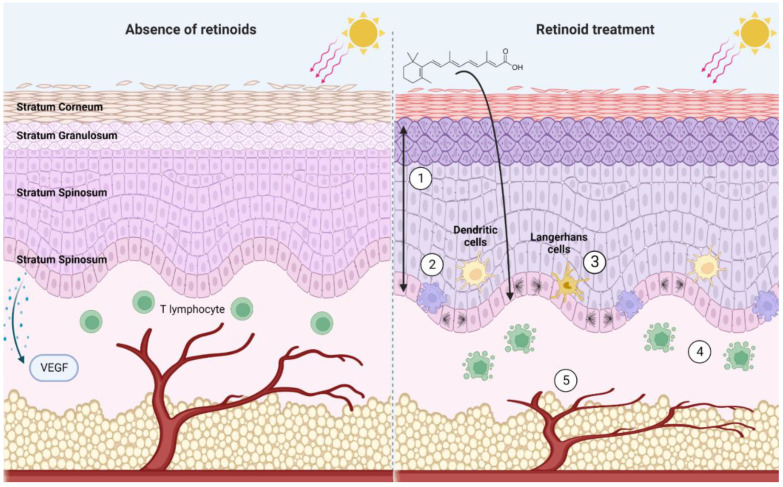
A summary of noteworthy effects of pharmacological retinoid signaling within the skin. In the absence of retinoids, UV radiation depletes the skin of Langerhans cells and dendritic cells. VEGF production by the basal keratinocytes is stimulated by UV radiation, stimulating angiogenesis. In the presence of retinoids (1) Upregulation of basal cell proliferation results in thickening of stratum spinosum and stratum granulosum, and a thinning of the stratum corneum. Cumulative increase in epidermal thickness (2) increased apoptosis of basal cells (3) dendritic cells and Langerhans cells are protected from UV-induced decrease in cell number (4) apoptosis of T cells (5) prevent VEGF release in response to UV irradiation, inhibiting angiogenesis. Created with BioRender.com.

These mechanisms (summarized in Figure 3) allow for retinoids to serve as effective chemoprophylactics and chemotherapeutics. Retinoids are master-regulators of cutaneous physiology, assuming control over processes that are integral to cancer pathogenesis. The following section highlights retinoid’s anticancer effects captured in experimental models of KCs.

## 4. The Molecular Mechanisms of Retinoid Activity in Experimental Models of Keratinocyte Carcinomas (KCs)

In experimental models of KCs, retinoids have demonstrated promise as chemoprophylactics and as chemotherapeutics. These models include cell lines, organotypic skin, murine models and patient-derived xenografts. Experimental models of KCs have enabled researchers to obtain insights into the molecular mechanisms underlying retinoid activity in skin cancer. Herein, we will describe how disruptions in retinoid signaling can contribute to skin cancer formation, as well as how retinoids have been shown to effect BCC, cSCC and other skin cancers in experimental models.

### 4.1. Oncogenic Disruptions in Retinoid Signaling Contribute to Skin Carcinogenesis

The retinoid signaling pathway is disrupted in a plethora of malignancies and these disruptions can be oncogenic [13]. Aberrant expression or function of RARs, RXRs, CRABPII, STRA6 and metabolic enzymes have been linked to the development of KCs and other skin cancers [97,99,154,155]. These oncogenic alterations generally impair retinoid signaling, either by reducing bioavailability or signal transduction [97,99,154,155]. The presence of oncogenic retinoid signaling aberrations is not the sole determinant of which cancers are amenable to retinoid therapies. Even for skin cancers in which the retinoid signaling axis is intact, retinoids elicit effects that are desirable for cancer prevention and treatment.

### 4.2. The Prophylactic and Therapeutic Benefits of Retinoids in BCC Are Attributed to Apoptosis and Modulation of Oncogenic Signaling Pathways

Experimental evidence supports the use of retinoids for BCC chemoprevention and treatment. So et al. conducted BCC chemoprevention studies using tazarotene [156]. Topical 0.1% tazarotene gel was effective at reducing the number and size of BCCs formed in UV- or ionizing radiation-exposed mice over time [156]. Studying BCC formation in a Patched 1 (Ptch1) −/+ mouse model representative of NBCCS, tazarotene effectively reduced BCC formation, even 5 months after treatment was ceased [156]. Other topical retinoids, including ATRA and a selective RARα agonist (AGN195813), were compared to tazarotene as BCC chemoprevention drugs in Ptch1 −/+ mice exposed to UV irradiation [157]. Tazarotene was superior to other retinoids at repressing microscopic and macroscopic BCC formation [157]. In murine models of BCC, topical 0.1% tazarotene was also capable of promoting tumor regression, suggesting a possible therapeutic benefit in humans [157].

Retinoids exert their anti-cancer effects on BCC cells by promoting apoptosis and regulating signaling pathways that are integral to BCC pathogenesis. Tazarotene promotes apoptosis of primary human BCC cells in vitro [158]. Mechanistically, apoptosis arises due to mitochondrial dysfunction, generation of reactive oxygen species (ROS), followed by cytochrome c release and induction of the caspase 8/t-BID cell death pathways [158]. Tazarotene also restores expression of TIG-3 in BCCs; an important apoptosis-regulatory protein that mediates microtubule redistribution and organelle accumulation during apoptosis [159]. Oncogenic signaling pathways that are hyperactivated in BCC may be repressed by retinoid treatment. The PTCH1-Gli signaling pathway is central to BCC pathogenesis [160]. Overactivation of PTCH1-Gli signaling promotes proliferation, cancer stemness and other tumorigenic phenotypes [160]. Treatment of murine keratinocytes and BCC cells with tazarotene significantly reduced Gli mRNA expression [157,161]. The PI3 kinase-AKT-mTOR pathway is also important for BCC pathogenesis, in part by dysregulating the cell cycle and promoting proliferation [162]. So et al. demonstrated that tazarotene represses overactivation PI3 kinase-AKT-mTOR signaling in murine BCCs [163]. These mechanisms represent possible ways whereby retinoids can restrict BCC tumor formation and maintenance.

### 4.3. Retinoids Suppress Several Hallmarks of Tumorigenesis in cSCC 

Retinoids are promising chemoprophylactic agents for cSCC. The effects of dietary and topical retinoid supplementation on the processes of chemical and UV-induced skin carcinogenesis were the subject of early preclinical studies [164,165]. The 7,12-Dimethylbenz[a]anthracene/12-Otetradecanoylphorbol-13-acetate (DMBA/TPA) two-step carcinogenesis mouse model is useful for the study of multi-stage cSCC tumorigenesis [166]. Dietary retinol, ATRA, fenretinide and β-carotene supplementation inhibits chemical carcinogenesis irrespective of the stage of malignant progression at the onset of treatment in the DMBA/TPA model [165,167,168,169]. It was reported that topical ATRA application reduces malignant conversion of benign skin papillomas to SCCs [170]. However, a subset of retinoid-resistant papillomas progress to develop aggressive disease [170]. Dietary 13-cis RA on its own or when combined with difluoromethylornithine (Eflornithine) could inhibit cSCC formation in mice exposed to TPA [171]. Similarly, oral fenretinide can repress DMBA/TPA-induced murine skin carcinogenesis, likely through a bioactive metabolite [84,172]. Another study used benzoyl peroxidase (BPO), a free radical-generating mutagen, to promote oxidative stress and skin tumorigenesis [173]. Pre-treatment with topical isotretinoin attenuated the accumulation of oxidative damage, impaired the mutagenic activity of BPO and reduced cSCC formation [173].

Several mechanisms may be involved in retinoid-repression of cSCC formation. Huang et al. investigated the ability of ATRA treatment to repress activator protein-1 (AP-1) activation as a mechanism of inhibiting cSCC formation [174]. AP-1 is a heterodimeric transcription factor that is overexpressed in several types of aggressive cancers and associated with malignant transformation [175,176]. Using an AP-1-luciferase reporter mice treated with DMBA/TPA, researchers reported that topical ATRA application suppressed tumorigenesis [174]. A synthetic retinoid that selectively activates transcription at the RARE without AP-1 repression was insufficient to suppress tumorigenesis. Conversely, a synthetic retinoid that represses AP-1 without activation of the RARE was sufficient to suppress tumorigenesis. Taken together, activation of the RARE may be dispensable for chemoprevention with ATRA, while AP-1 repression may be necessary [174]. Syed et al. proposed that ATRA can repress cSCC formation by downregulating STAT3 activation in keratinocytes [177]. STAT3 activation promotes keratinocyte survival and proliferation, and is implicated in malignant progression [178,179]. A study conducted in premalignant HaCaT cells treated with acitretin corroborates these findings [115]. Another proposal is that retinoids confer protection against UV damage. The spectral properties of retinoids reduce UV-induced DNA damage [180]. Similarly, exposure of murine skin to UV radiation promotes a local decrease in RARγ and RXRα, ablation of which can promote cSCC formation [97,119,181]. Retinoid pre-treatment prevents the UV-induced decrease in levels of retinoid receptors [181]. Interestingly, retinoid treatment can also regulate angiogenesis, a process that is necessary for tumor establishment and maintenance [182]. Acitretin treatment restricted tumor angiogenesis in a cSCC xenograft established using A431 cells, as well as murine papilloma cells and HPV-transformed keratinocytes [183]. Hence, angiogenesis inhibition may represent another mechanism by which retinoids suppress skin cancer formation.

In addition to chemoprevention, retinoids exhibit potential as cSCC therapeutics, through their ability to control proliferation and the cell cycle. Treatment with retinoids can inhibit cSCC proliferation [184]. Coupling retinoid treatment with interferon α (IFN-α) may further potentiate this antiproliferative effect in the human A431 cSCC cell line [184]. ATRA treatment promotes G1 cell cycle arrest in the SCL-1 cell line [185]. This arrest was marked by decreases in the expression of cyclin D1/CDK4 as well as increased levels of p21/Waf1 and p27/Kip1 proteins [185]. This anti-proliferation effect may be caused by ATRA-induced downregulation the MAPK pathway [185] The ability of retinoids to downregulate the MAPK pathway was later recaptured in the DMBA/TPA murine model of cSCC [186].

**Figure 4:**
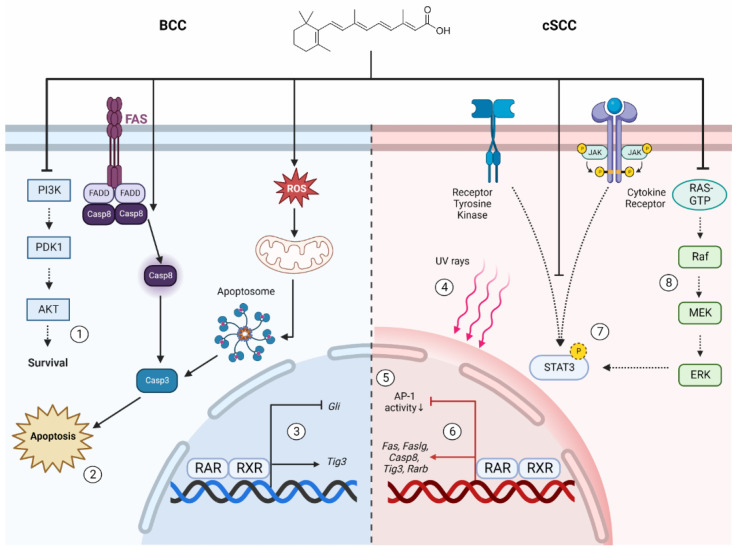
Molecular mechanisms of retinoid action in keratinocyte carcinomas. BCC (**left**) and cSCC (**right**). Retinoids have been shown to (1) downregulate PI3K signaling, (2) promote apoptosis through regulation of the extrinsic death receptor pathway and mitochondrial cell death pathway (3) suppression of Gli transcription to limit PTCH1 pathway activation as well as increase in Tig3 tumor suppressor transcription. In cSCC, (4) spectral properties of retinoids protect against UV damage (5) decrease Ap-1 activation (6) promote proapoptotic (Fas, Faslg, Tig3, Casp8) and tumor suppressor (Rarb) gene expression (7) inhibit oncogenic STAT3 activation (8) downregulate Ras-Raf-MEK-ERK signaling. Figure created using Biorender.com.

Retinoid signaling also promotes cSCC cell apoptosis. Lin et al. investigated the effect of acitretin on the human cSCC cell line SCL-1 [187]. Acitretin reduced the viability of SCL-1 cells, but not that of premalignant HaCaT cells [187]. Acitretin induces SCL-1 apoptosis through the extrinsic cell death pathway, by increasing expression of Fas (CD95), Fas ligand and caspase-8 mRNA [187]. TIG-3 induction may also represent a mechanism of retinoid induced apoptosis in cSCC cells [159]. Fenretinide was shown to be a potent inducer of apoptosis in A431 cSCC cells in vitro [188,189]. The mechanism of fenretinide-induced apoptosis was proposed to be mediated by a metabolite intermediate, and may not depend upon RAR-RXR signaling [84,190]. To summarize, retinoids exhibit the capacity to suppress cSCC tumorigenesis via: AP-1 and STAT3 repression, UV protection and angiogenesis restriction. Induction of cell cycle arrest and apoptosis may represent mechanisms by which retinoids can be used therapeutically to treat cSCC. Key anti-cancer effects of retinoids in keratinocyte carcinomas are illustrated in Figure 4.

### 4.4. Retinoids Exert Diverse Antineoplastic Effects in Models Representative of Other Skin Cancers

The ability of retinoids (and “rexinoids” in particular) to induce cell cycle arrest and apoptosis of malignant T cells forms the foundation for their use to treat different forms of CTCL [191]. Bexarotene induces apoptosis in multiple CTCL cell lines including MJ, HH and Hut78, marked by increased levels of cleaved caspase-3 and cleaved-PARP along with decreased survivin (anti-apoptotic protein) [192]. These effects may be partially explained by activation of p53 and upregulation of p73 expression, which was observed in a later study conducted on the same cell lines [193]. In addition to bexarotene, alitretinoin (9-cis RA) was investigated in vitro for the treatment of CTCL using cell lines [194]. Alitretinoin resulted in dose-dependent decrease in cell viability of Hut78 and MyLa 2059 cells, stimulating apoptosis and increasing the proportion of cells in G0/G1 [194,195]. The authors determined that alitretinoin treatment impaired STAT3 and STAT5 phosphorylation in CTCL cell lines, presumably by decreasing activation of the JAK/STAT signaling pathway while promoting activation of the RAR/RXR pathway [194].

**Figure 5:**
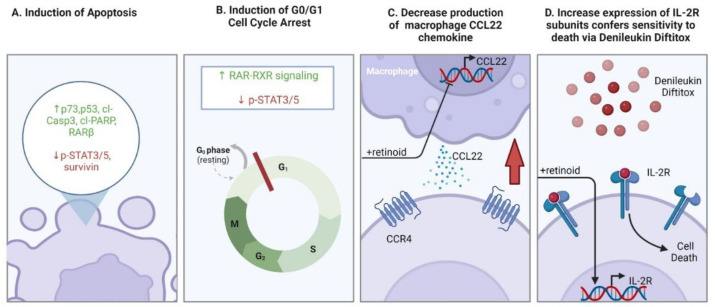
A Summary of retinoid action in CTCL cells. (**A**) Retinoids promote apoptosis, indicated by an increase (green text) in p73, p53. PARP cleavage and Caspase-3 cleavage are evident. Downregulation of STAT3/5 phosphorylation and expression of anti-apoptotic survivin protein (red). (**B**) cell cycle arrest in G0/G1 has been attributed to increased RAR-RXR signaling and decrease p-STAT3/5. (**C**) Decreasing production of CCL22 from tumor associated macrophages, thereby limiting malignant T cell recruitment into the skin. (**D**) upregulating expression of IL-2R subunits sensitizes cells to cell death using the fusion protein denileukin diftitox. Figure created with Biorender.com.

Regulation of apoptosis and cell cycle are not the only mechanisms by which retinoids influence CTCL pathogenesis. Co-treatment using a retinoid (Am80) and a histone deacetylase inhibitor induced expression of the tumor suppressor RARβ in a CTCL xenograft model established using the SeAx cell line [196]. Retinoids also regulate chemotaxis of malignant T cells in the skin. SS cells treated with bexarotene in vitro downregulated the skin-homing chemokine receptor, C-C chemokine receptor type 4 (CCR4) [197]. CCR4^+^ cells can include regulatory T lymphocytes (Tregs) and type II helper T lymphocytes (Th2) [198]. A study by Tanita et al. found that tumor-associated macrophages in CTCL lesions produce the chemokine CCL22 [198]. CCL22 recruits CCR4-expressing T lymphocytes into the skin [199]. CCL22 expression in the microenvironment is increased in advanced-stage CTCL compared to early stage [198]. Bexarotene treatment reduced production of CCL22 by macrophages [198]. Therefore, recruitment of CCR4^+^ lymphocytes into the skin of CTCL patients could be reduced by bexarotene treatment, though this remains to be demonstrated in a clinical research study. A recently proposed molecular subtype of CTCL identified through single cell transcriptomics was determined to have a higher percentage of M2 macrophage infiltration [200]. This subtype of CTCL was proposed to be most amenable to retinoid therapy because of bexarotene’s ability to modulate the tumor microenvironment [200]. Bexarotene and alitretinoin were also found to exert immunomodulatory effects upregulating the expression of cell-surface interleukin-2 receptor (IL-2R) subunits in Hut78 and Hut102 CTCL cells [201]. This upregulation increased susceptibility of these cells to the fusion protein, denileukin diftitox [201]. Co-treatment of cells with bexarotene and denileukin diftitox was more cytotoxic to CTCL cells compared to either agent alone [201]. The mechanisms of retinoid action in CTCL are depicted in Figure 5.

Experimental models reveal that retinoids may also be beneficial for treating KS. Corbeil et al. investigated the impact of various retinoids on the biology of KS cells, including tretinoin, acitretin, isotretinoin and the synthetic retinoid RO13-1470 [202]. Established KS cell lines were treated with low or high doses of retinoids. Low-dose retinoids exerted an anti-proliferative effect, whereas high-dose retinoids induced apoptosis [202]. These effects were hypothesized to be mediated by changes in interleukin-6 production (IL-6) [202]. IL-6 acts as an autocrine stimulatory factor for KS cells [203]. Using KS cell lines, Nagpal et al. found that ATRA treatment suppressed proliferation of KS cells and suppressed IL-6 production through direct activity of RAR-RXR heterodimers at enhancer sites for the transcription factor NF-IL6 [204]. The third-generation retinoid, fenretinide, has also been explored as a therapeutic modality for KS in vivo. Mice implanted with KS xenografts were given oral fenretinide [205]. Fenretinide decreased tumor growth relative to vehicle [205]. In an in vitro angiogenesis assay, the fenretinide treatment also decreased tumor cell-induced angiogenesis, which is particularly important for KS tumors [205]. These results suggest that retinoids are an effective treatment for KS. 

## 5. Clinical Applications of Retinoids for Keratinocyte Carcinoma Chemoprevention

Epidemiological studies illustrating inverse correlations between retinol intake and cancer incidence have supported the use of retinoids as chemoprophylactics in humans [206,207]. Retinoid chemoprevention studies have centered primarily on suppressing KC formation in high-risk patients. High-risk patients include those who have developed multiple KCs, immunosuppressed patients and or those who are afflicted with genodermatoses that confer KC predisposition (e.g., NBCCS) [22]. Several clinical trials have been conducted to assess the efficacy and safety of retinoid chemoprevention among different cohorts of patients who are at high-risk of developing skin cancers. Some retinoids are used off-label as KC chemoprophylactics, and the use of retinoids for skin cancer prevention (namely for cSCC prevention) is recommended in guidelines outlined by the U.S. National Comprehensive Cancer Network (NCCN) [10,11,208].

Retinoids are effective at preventing the formation of KCs in immunosuppressed SOTR [209,210]. Bavinck et al. performed the first randomized placebo controlled trial assessing oral acitretin (30 mg/day) for the chemoprevention of KCs in renal transplant recipients. At the end of their study, conducted across 6 months, patients in the acitretin treatment arm had developed fewer KCs and premalignant lesions (AKs) compared to the placebo arm [209]. Another study assessed low-dose (0.2 mg/kg/day) acitretin for AK and KC chemoprevention in renal transplant patients [211]. Whilst the low-maintenance dose could reduce size and incidence of AK lesions, malignant tumor incidence was unchanged [211]. Harwood et al. performed a 16-year retrospective study recording the incidence of SCCs before and after solid organ transplantation in patients started on low-dose of acitretin for at least one year [210]. In the first three years after beginning retinoid therapy, SOTR patients developed fewer cSCCs compared to before starting retinoid treatment [210]. This prophylactic effect persisted for up to 8 years [210]. These benefits were imparted in the absence of significant hepatic, renal or skeletal toxicities. Mucocutaneous xerosis was commonly observed, as were elevations in triglyceride and cholesterol levels, necessitating lipid-lowering therapy. The researchers did not report any adverse cardiovascular outcomes, nor did they cite any episodes of triglyceride-induced pancreatitis [176]. A later study reported that the incidence of BCC in addition to cSCC/cSCC in situ (Bowen’s disease) was decreased in SOTR after the start of acitretin therapy compared to before [212]. While not FDA approved at this time, use of acitretin for KC and AK chemoprevention in SOTR is supported by evidence, and even recommended by the NCCN (for cSCC) [208].

In addition to preventing skin cancer formation in patients receiving iatrogenic immunosuppression, retinoids can be useful to prevent skin cancer formation in patients receiving treatments that increase cancer risk. Nijsten et al. conducted a nested cohort study among psoriasis patients treated with psoralen-UVA (PUVA) therapy taking oral retinoids [213]. Psoriasis patients receiving PUVA therapy have an increased risk of developing cSCC [214]. Oral retinoid use was associated with a lower incidence of cSCC among patients who have received PUVA therapy for psoriasis [213]. Another study assessed oral acitretin as a means of preventing cSCC formation in stage IV melanoma patients receiving vemurafenib or dabrafenib monotherapy [215,216,217]. Patients taking these BRAF inhibitors are at an increased risk of developing cSCC and other skin tumors [215]. In a cohort of 8 patients, only 5 cSCC tumors developed in two patients after the start of therapy compared to 24 cSCC tumors across all 8 patients before the start of acitretin therapy [215]. These studies support the use of retinoids to suppress iatrogenic cSCC formation. The use of retinoids as an adjuvant to surgery for KC, has not been explored extensively. One study assessed oral isotretinoin combined with subcutaneous injection of IFN-αA2 as an adjuvant therapy following surgical resection or irradiation of advanced cSCC, to prevent formation of second primary tumors [218]. However, this adjuvant therapy was deemed ineffective, and the prospect of using this combination as secondary chemoprevention after tumor resection was abandoned [218]. 

Patients with high rates of KC tumor formation may be amenable to chemoprevention with systemic retinoids. A randomized controlled trial conducted by Kadakia et al. supports the use of oral acitretin for immunocompetent patients with high rates of KC (≥2 tumors within a span of five years) [219]. Similar to findings reported by Harwood et al., oral acitretin was most commonly associated with mucositis, alopecia and other skin disturbances compared to placebo treatment. Furthermore, hypertriglyceridemia and liver function test (LFT) elevations were found to be of low severity and non-significant between treatment arms [182]. Topical agents have proven less successful in clinical trials. Topical tretinoin was frequently used off-label for KC prevention [220]. Weinstock et al. conducted The Veterans Affairs Randomized Chemoprevention Trial [220]. Veterans are considered to be at high-risk for developing skin cancers provided high levels of occupational sun exposure [221]. Over 1000 patients were recruited from the veteran population and placed on 0.1% topical tretinoin or matched vehicle formulation for up to 5.5 years [220]. The primary endpoint was the formation of new BCCs, SCCs, or AKs on the face or ears. Ultimately, 0.1% topical tretinoin was ineffective at reducing the formation of BCC, SCC and AKs [220]. Nonetheless, off-label use of retinoids as chemoprophylactic measures in patients with multiple KC tumors is acceptable provided an abundance of successful reports [10,11].

Patients with cancer-predisposing genodermatoses benefit from retinoid chemoprevention. One clinical trial reported that treatment with isotretinoin reduced the formation of BCCs and SCCs in XP patients [222]. After discontinuing treatment, tumor formation increased by 8.5 fold [222]. Side effects mainly pertained to significant but non-limiting xerosis and conjunctivitis, which lessened as treatment progressed. LFTs had transient increases during initiation of therapy but returned to normal limits as treatment progressed for all but one patient, who required withdrawal from the study due to persistent elevation. Triglyceride levels followed a similar pattern and could be managed with diet modification. Asymptomatic ligament calcification was also noted in two patients involving either the spine or feet [185]. Case reports detailing suppression in skin tumor formation in XP patients taking etretinate have been described [223,224]. Oral isotretinoin has also been proposed as a chemoprophylactic agent for NBCCS [225,226]. Furthermore, 0.1% topical tazarotene cream was investigated for BCC suppression in NBCCS patients [227]. This study followed a randomized, double-blind vehicle controlled trial [227]. The primary endpoint for chemoprevention was the number of back, chest and face BCCs that developed after two years of tazarotene treatment compared to the first year before treatment [227]. Only 6% of patients exhibited a partial or complete reduction in skin cancer formation [227]. Hence, it was deemed that, despite strong preclinical evidence, topical tazarotene is not an effective means of BCC chemoprevention in NBCCS patients. Retinoids have also been used to prevent malignancy in other skin cancer-predisposing genodermatoses, such as Muir-Torre syndrome and Bazex Syndrome [10,11,228]. Overall, chemoprevention with oral isotretinoin remains the primary off-label prophylactic for NBCCS and XP patients [10].

To summarize, retinoids are not currently FDA-approved for the chemoprevention of KCs. Nevertheless, substantial evidence demonstrating that these compounds are useful skin cancer prophylactics has promoted their off-label use for skin cancer prevention in high-risk patients [10]. Acitretin has exhibited efficacy in clinical trials as a prophylactic agent for AKs and KCs in SOTR and in other settings where the risk of skin cancer development is high. Oral isotretinoin is also used as a KC prophylactic for XP and NBCCS patients. The off-label use of oral retinoids for chemoprevention in these contexts is widely-accepted and recommended by experts [208].

## 6. The Use of Retinoids for Skin Cancer Treatment

Retinoids are effective treatments for some skin cancers [10,11]. BCC and cSCC were among the first malignancies to be treated with retinoids [14]. Despite theoretical support, experimental evidence and an abundance of encouraging case reports, large-scale clinical trials have suggested that retinoids are ineffective treatments for the majority of KCs (once the tumor has formed), whereas topical and systemic retinoid therapies are effective treatments for other primary cutaneous malignancies, namely CTCL and KS [11]. Some indications are FDA-approved [10,11]. Herein, we describe the use of retinoids for the treatment of KCs and other skin cancers. 

### 6.1. The Prospect of Retinoids to Treat Keratinocyte Carcinomas and Premalignant Lesions

The first successful application of retinoids as a cancer treatment was for a case of BCC [229]. Case reports have since been published detailing the use of both systemic and topical retinoids to treat BCC tumors [230,231]. In a preliminary study by Peck et al., oral isotretinoin (~3 mg/kg/day) only induced regression in 8% of studied BCC tumors [225]. The high dose of isotretinoin was notably associated with morbid skeletal toxicity, in conjunction with significant elevation in triglyceride and LFT laboratory results [225]. Lower doses (0.25–1 mg/kg/day) reduced the rate of adverse events but were deemed ineffective as chemotherapeutic treatment options. A study by Hughes et al. provided more encouraging results, investigating etretinate as a therapy for AKs and BCCs. Patients were administered oral etretinate (1.5 mg/kg/day for the first month, then 0.75 mg/kg/day for two months thereafter) and followed from 12 weeks to 18 months with regular visits in between [232]. Both the number and size of AK and BCC lesions were decreased post-treatment [232]. Additionally, it was observed that etretinate treatment altered the expression of histological and biochemical markers of metabolism in BCC cells but not adjacent normal skin [232]. The adverse event profile was also unremarkable, with transient hypertriglyceridemia returning to baseline following cessation of therapy being cited as the most notable toxicity [202]. Topical retinoids have been proposed to treat BCC as well. Topical 0.05% tretinoin was used effectively in an early study to regress BCCs, but there was a high rate of recurrence after treatment had ceased [233]. Tazarotene creams exhibited greater promise in clinical studies. Peris et al. observed in one study that 53% of nodular or superficial BCC patients treated with topical tazarotene experienced complete responses, offering promising preliminary evidence [234]. Duvic et al. interrogated the use of 0.1% topical tazarotene for the treatment of BCC tumors [235]. Almost 60% of tumors treated with tazarotene for 12 weeks had regressed prior to excision. Mild-to-moderate local symptoms were reported, including crusting/scabbing, erythema and ulcerations. Hence, tazarotene cream confers an appreciable level of clinical benefit [235]. Clinical trials to further evaluate tazarotene as a BCC therapy have not been conducted.

Premalignant AKs and in situ cSCC (known as Bowen’s disease (BD)) have been successfully treated with retinoids. Ianhez et al. provided a comprehensive review of studies investigating the use of retinoids for AK treatment [236]. Clinical trials have investigated both systemic and topical agents to clear AKs, including retinoids representing different generations [236]. Results have been mixed, with clinical trials reporting high efficacy in some studies, and low efficacy for others. Nonetheless, considerable evidence supports the use of topical tretinoin and adapalene for AK treatment. Specifically, off-label use of 0.1% topical tretinoin as well as 0.1–0.3% topical adapalene may be considered beneficial to treat AKs [11,14,220,237]. Combination treatments to treat AKs may be more robust compared to retinoid monotherapy. Notably, retinoids combined with topical or intralesional 5-fluorouracil has been proposed as an effective intervention [238]. A recent study investigated photodynamic therapy with adapalene pretreatment to regress AKs of the upper extremities [239]. This combination therapy was highly effective at regressing AK lesions [239]. In addition to AKs, Bowen’s disease lesions may be amenable to retinoid therapy. One pilot study assessed 0.1% topical tazarotene applied once daily for the treatment of Bowen’s disease in 15 patients [240]. Of the 10 patients who completed the 6-month treatment course, 7 had complete responses and 3 had partial responses, clinically and histologically [240]. The regression was sustained at the 3-month follow-up and the treatment was well-tolerated by most patients [240]. These interventions have not been evaluated in large clinical trials.

Early studies in the 1980s contemplated the use of oral isotretinoin for cSCC treatment. Oral isotretinoin (1–2 mg/kg/day) was an effective intervention for a subset of cSCC patients with advanced disease [241,242]. Additionally, the combination of isotretinoin capsules with subcutaneous injection of IFN-α2A was found to be effective for some patients with advanced cSCC [243]. A recent case report detailed the successful use of oral acitretin in combination with clarithromycin to treat cSCC in three patients [244]. Furthermore, the researchers did not note any significant increases in toxicities despite an established interaction between clarithromycin and acitretin via CYP3A4 inhibition [244]. Other retinoids have not been investigated in clinical studies for cSCC treatment. There is a precedent to investigate third and fourth generation retinoids for cSCC treatment, provided their desirable effects in experimental studies. 

Despite substantial preclinical evidence and an abundance of case reports documenting the use of retinoids to treat BCC and cSCC, retinoids have failed to exhibit appreciable efficacy, tolerability and a sustained response in large-scale clinical trials. Retinoids are not currently FDA-approved for the treatment of KCs, and are not currently recommended for off-label uses [11]. Topical tretinoin exhibits efficacy for treating AKs, but it is not FDA-approved for this use [11]. Nonetheless, the off-label use of topical tretinoin is a generally accepted for AK treatment [11]. 

### 6.2. Retinoids Are Effective Treatments for CTCL and KS

Several retinoids and, especially, rexinoids, have been investigated as treatments for CTCL, as monotherapies and in combination with other therapies. Oral isotretinoin (1–2 mg/kg/day) was first considered for MF treatment, introducing retinoids as a possible effective and tolerable treatment modality [195,245,246,247,248,249] with minimal associated toxicities [206,207]. These initial efforts were followed by studies evaluating oral etretinate therapy. Etretinate as a monotherapy and in combination with electron beam therapy exhibited efficacy for the treatment of MF [250,251]. However, etretinate was taken off the market and replaced with acitretin, which does not persist in the body’s tissues for as long as etretinate [10]. A retrospective chart review contemplated the use of oral acitretin, as a monotherapy or in combination with other treatments, to manage, MF, SS and a case of unspecified CTCL [252]. Anecdotal evidence of the successful use of acitretin to treat CTCL had been documented previously [252]. Based on their analyses, the authors concluded that acitretin might achieve appreciable response rates, when compared to standard therapies, with a tolerable safety profile, although other retinoids were shown to have higher efficacy [252]. Clinical trials to evaluate acitretin as a therapy for CTCL have not been conducted, and nor have preclinical studies to determine the biological effects of acitretin on CTCL cells.

Third-generation retinoids have been used as successful therapies for CTCL. Duvic et al. investigated oral bexarotene capsules as therapy for advanced-stage (IIB-IVB) and refractory CTCL (both MF and SS variants) [253]. Phase II/III clinical trials reported 45% complete and partial responses in patients assigned to a starting dose of 300 mg/m^2^/day. Notably, 55% of patients who received a higher dose had complete or partial responses. The most notable side effect was hypertriglyceridemia-induced pancreatitis which did not re-occur following strict lipid management. Over 10% of patients exhibited a dose-dependent increase in LFTs. Importantly, 40% and 28% of patients developed central hypothyroidism and leukopenia, respectively [253]. Bexarotene capsules were also deemed effective and tolerable for refractory or persistent early stage MF [254]. Results of these trials were corroborated by those conducted in other countries [255,256]. Topical bexarotene formulations were also deemed effective and tolerable in clinical trials. Breneman et al. conducted phase I and II clinical trials assessing the efficacy of bexarotene gel formulations ranging from 0.1–1.0% for MF, presenting encouraging results [257]. Phase III trials would substantiate these findings; topical bexarotene was deemed an effective treatment for refractory and persistent early stage MF [258]. One important barrier of using this product is its prohibitive cost [259].

Optimal use of retinoids for cancer treatment may require a combination therapy [12]. The HDAC inhibitor vorinostat, UVB-NB phototherapy, PUVA therapy, methotrexate and the fusion protein denileukin diftitox have all been subject to clinical trials in combination with bexarotene [260,261,262,263,264]. In a phase III clinical trial, the combination of bexarotene with PUVA therapy had similar efficacy to PUVA alone (77% complete or partial response compared to 71% for PUVA alone), but allowed for a lower dosage of PUVA to be effective [265]. A phase I clinical trial also supported bexarotene tablets in combination with denileukin diftitox infusion for patients who had disease progression after a failure of first-line therapies [263]. The authors determined that the combination was effective (67% complete or partial responses) and tolerable, even with lower doses of bexarotene [263]. Non-dose dependent hypertriglyceridemia was also observed with the lowest administered doses of bexarotene. However, no incidences of pancreatitis were reported. Furthermore, similar to the Phase II/III clinical trials [211], hypothyroidism (64%) and lymphopenia (57%) were also noted [220].

Aside from bexarotene, there is a precedent to further investigate the use of other retinoid compounds to treat CTCL. One retrospective analysis examined the efficacy of oral alitretinoin for the treatment of MF and SS [266,267]. In one of the analyses 40 MF and 8 SS patients were recruited and were treated with 30 mg dose of oral alitretinoin achieving moderate success [266]. Based on these findings, Alitretinoin is used in select counties where bexarotene is not available (e.g., Canada) as an off-label treatment for CTCL [267]. The cost of alitretinoin is significantly cheaper in comparison to bexarotene.

Alitretinoin topical gel 0.1% as a monotherapy and as a component of combination therapy has been deemed effective for the treatment of CTCL [266]. Furthermore, a prospective open-label study evaluating the efficacy of 0.1% tazarotene gel for early stage MF reported 60% of patients demonstrating a complete response and 20% of patients having stable disease (the remaining patients stopped therapy early due to local side effects) [268]. Similarly, an open-label pilot study also assessed 0.1% tazarotene gel for refractory MF [269]. This study reported that 58% of patients enrolled in their study observed a reduction of at least 50% in the body surface area involvement. In skin biopsies, malignant lymphocytic infiltration of the skin was decreased by tazarotene treatment, whilst CD8^+^ antitumor T cell counts were elevated, suggestive of a higher immune response activation [269]. 

Based on these trials, topical bexarotene gel in formulations up to 0.1%, as well as capsules, have been FDA-approved for the treatment of early stage and refractory MF as a secondary treatment option after other avenues of treatment have been exhausted [11]. Other retinoid formulations have not been investigated for CTCL treatment. One completed clinical trial investigating trifarotene for early stage CTCL has not reported results at this time [148]. Off-label use of topical tazarotene for CTCL has shown moderate success [11]. 

KS lesions are also well-managed with retinoids. Oral ATRA alone or in combination with IFN-α2a was tolerable but ineffective at producing a robust anti-tumor effect for AIDS-related KS [270]. Phase I and II trials reported that topical alitretinoin treatment was well-tolerated and effective as a monotherapy, and compatible with standard anti-retroviral therapies taken concurrently [271,272]. Phase III trials further substantiated the use of alitretinoin gel for KS lesions, with an overall response rate of 37% [273]. Alitretinoin capsules provided a moderate and durable effect, but caused appreciable side effects at high doses that limited their use [274]. Supported by these trials, topical 0.1% alitretinoin gel has been FDA-approved for the treatment of AIDS-related KS [11]. Notably, 0.1% alitretinoin is considered for use off-label to treat non-AIDS related KS, based on the documented success [275]. Oral acitretin has also been used to treat classic KS (unrelated to HIV) successfully, although not typically used clinically [276]. 

In summary, retinoids are useful prophylactic and treatment agents for skin cancers with select FDA-approved and off-label applications. Generally, these compounds are well-tolerated, although monitoring for side effects and adverse events (i.e., local skin reactions for topical agents, metabolic/endocrine changes for systemic agents, musculoskeletal and neurological events) is critical [10]. These compounds are notoriously teratogenic [277]. Hence, pregnancy prevention measures and screening is of particular importance [10]. The clinical uses of retinoids for skin cancer treatment and prevention are summarized in Table 1. 

## 7. Conclusions and Perspectives

Since their discovery, retinoids have gained appreciation as potent and versatile dermatological therapies. Despite decades of biochemical and clinical investigation, several challenges remain in the efforts to use retinoids for skin cancer prevention and treatment. First, there are novel retinoid compounds that have not been subject to extensive testing for their use in skin cancer (and especially KC) management. Notably, trifarotene (which is FDA-approved for acne vulgaris under the trade name Aklief^®^ [Galderma]) has not been assessed in experimental models for the treatment of KCs [148]. Trifarotene is highly selective for RARγ, possesses anti-inflammatory properties and controls processes implicated in skin cancer pathogenesis (e.g., differentiation, proliferation, etc.) [58]. Furthermore, this drug is metabolised relatively efficiently in the liver and is well-tolerated [58]. Thus, future studies investigating how trifarotene may be used to manage skin cancers are justified [148].

Second, there are knowledge gaps between basic research findings and clinical translations. As described, several retinoid compounds have exhibited promise for KC/skin cancer prevention and treatment in experimental settings. Nonetheless, many of these compounds, have not been studied in human subjects nor in clinical trials at this time. This is especially true for third- and fourth-generation retinoids, which have demonstrated considerable promise as cancer therapeutics. The use of retinoids as a component of combination therapy has also long been suggested, but hardly investigated in dermatology research [12]. Rational drug combinations featuring retinoids may be critical to managing side effects and optimizing the use of these compounds for skin cancer management.

Third, retinoids have short-lived effects and they do incur side effects that can limit tolerability and clinical applications [10,11]. Adverse events associated with retinoid compounds, such as xerosis, hyperlipidemia/hypertriglyceridemia, hypothyroidism (central), hepatic dysfunction and skeletal toxicities can necessitate discontinuation of retinoid therapy. There is a persistent need to optimize retinoid formulations and therapeutic strategies to minimize off-target effects, maximize tolerability and ensure that effects are sustained. Optimizing retinoid drug delivery systems has been postulated as a means of maximizing efficacy, durability of response and specificity [280]. Another point of concern is the possibility of drug interactions influencing the safe use of retinoids in individuals taking medications to treat unrelated diseases. For example, CYP3A4 inhibitors can raise the incidence of retinoid-induced toxicities [10] yet are commonplace in many therapeutic regimens. Furthermore, the effect of retinoids on hepatic function, which decreases moderately throughout adulthood, has not fully been elucidated. Provided the known hepatic risks of retinoid usage in healthy young patients and the relatively aged demographic of KC /skin cancer patients, retinoid usage at chemotherapeutic doses may cause additional hepatotoxicities for elderly patients.

There is an emerging body of research dedicated to understanding the molecular underpinnings of retinoid-responses and resistance in cancer cells [13]. Several mechanisms by which malignant cells become unresponsive to retinoids have been documented [13]. Retinoid metabolism pathways can be altered [59]. Cancer-specific gene expression can lead to the overexpression of factors that may confer retinoid resistance (e.g., the ectopic expression of the retinoid repressor preferentially expressed antigen of melanoma, PRAME, within cancer cells) [13,281]. Therapeutic biomarkers to gauge retinoid response and predict resistance could, therefore, be a promising focus for future studies, possibly allowing for the identification of patients most likely to respond to retinoid therapies. Co-targeting molecular determinants of retinoid sensitivity/resistance may improve the efficacy of retinoid compounds, and assist in offsetting adverse events. Effectively, characterizing the molecular genetics of retinoid response, and using this knowledge to optimize patient selection, design treatment strategies and infer prognosis can be a fruitful avenue of research to pursue. Such research, ushering the age of precision medicine, can help guide the future use of retinoids in dermatology.

## Figures and Tables

**Table 1:** FDA-approved and acceptable off-label applications of retinoids to treat and prevent skin cancer formation. Proposed mechanisms of action and adverse events included. Abbreviations: AK, Actinic Keratoses; AIDS, Acquired Immunodeficiency Syndrome; CTCL, Cutaneous T-Cell Lympoma; KS, Karposi Sarcoma; MF, Mycosis Fungoides; SOTR, Solid Organ Transplant Recipients.

Name (s)	Trade Names (Manufacturer)	Generation	FDA-Approved Uses for Skin Cancer Management	Off-Label Uses for Skin Cancer Management	Proposed Mechanisms of Action	Adverse Events/Warnings	References
Acitretin	Soriatane (Roche)	Second	None	Oral dosing for KC and AK chemoprevention in SOTR and other at-risk individuals.	High affinity for CRABPII displaces RA and potentiates endogenous RA signaling.	Teratogenicity skeletal abnormalities Hypertriglyceridemia Hyperlipidemia Hepatotoxicity Xerosis	[10,76,209,211]
Adapalene	Differin (Galderma)	Third	None	0.1–0.3% topical formulation for AK treatment	Selective RARγ agonist DNA damage and S-phase arrest in immortalized keratinocytes	Irritation, erythema, burning	[11,113,237]
Alitretinoin 9-cis retinoic acid	Toctino (Basilea)	First	0.1% topical formulation for AIDS-related KS	0.1% topical formulation for non-AIDS related KS. 0.1% topical formulation for CTCL second-line therapy. Oral dosing of 10–30 mg daily used off label for the treatment of CTCL.	RAR-RXR activation Induction of apoptosis in KS cells. Angiogenesis inhibition Inhibition of KSHV replication. G0/G1 cell cycle arrest, apoptosis and modulation of the JAK/STAT pathway.	Teratogenicity Irritation Dermatitis Scaling	[11,202,204,273,275]
Bexarotene Targretin^®^	Targretin (Ligand)	First	Oral dosing and topical 0.1–1.0% for advance-stage or treatment refractory MF resistant to at least one therapy.	None	Pan-RXR agonist Induction of apoptosis via p53/p73 activation Possible control over skin infiltration colonization by T cells	Teratogenicity irritation, erythema, burning dermatitis	[10,11,197,198,253]
Isotretinoin 13-cis retinoic acid Accutane^®^	Accutane (Roche), Claravis (Barr), Sotret (Ranbaxy), Amnesteem (Bertek), Absorica (Ranbaxy), Myorisan (VersaPharm)	First	None	Oral dosing for BCC/cSCC chemoprevention in NBCCS and XP.	RAR-RXR activation	Teratogenicity Skeletal abnormalities Hepatotoxicity	[10,11,225,226,278]
Tazarotene Tazorac^®^	Tazorac (Almirall)	Third	None	0.1% topical formulation for MF treatment as second line therapy	Selective RARγ agonist	Teratogenicity Irritation, erythema, burning	[11,268,269]
Tretinoin, All-trans retinoic acid (ATRA)	Vesanoid (Roche)	First	None	0.1% topical tretinoin to treat AKs.	RAR-RXR activation Induction of keratinocyte apoptosis. Control of hyperproliferative STAT signaling UV protection	Teratogenicity Irritation, erythema	[11,130,131,177,181,279]
